# Influence of baking conditions on the extractability and immunochemical detection of wheat gluten proteins

**DOI:** 10.1016/j.crfs.2022.100431

**Published:** 2022-12-30

**Authors:** Tanja Miriam Schirmer, Katharina Anne Scherf

**Affiliations:** a Leibniz-Institute for Food Systems Biology at the Technical University of Munich, Lise-Meitner-Str. 34, 85354 Freising, Germany; b Department of Bioactive and Functional Food Chemistry, Institute of Applied Biosciences, Karlsruhe Institute of Technology (KIT), Adenauerring 20 a, 76131, Karlsruhe, Germany

**Keywords:** Celiac disease, Crosslink, Enzyme-linked immunosorbent assay (ELISA), Food processing, Food safety, Protein extraction

## Abstract

Food processing conditions affect the accurate detection of gluten by ELISA, which is of importance for proper gluten-free labelling. We prepared different wheat flour-based and incurred baked goods (bread, crispbread, pretzel) to investigate the influence of baking conditions and alkali treatment on gluten quantitation by ELISA using different extraction solvents. Protein composition and extractability were determined (SDS-PAGE, RP-HPLC, GP-HPLC). The extraction solvents showed different performances; none of them could compensate the effect of baking on the detection. Dough preparation, baking and additional alkali treatment decreased protein extractability under reducing and non-reducing conditions. High temperature combined with alkali treatment resulted in the lowest protein extractabilities (<77% for bread crust, <61% for pretzel crust) due to the formation of disulfide and non-disulfide gluten crosslinks. There was no clear correlation between the protein composition and the extractability of alcohol- and SDS-soluble proteins of the baked goods. Thus, this research shows that gluten extractability rather than gluten composition is crucial for detection by ELISA in baked goods.

## Highlights

• Food processing including baking impairs gluten detection by ELISA.
• Common extraction solvents for ELISA do not achieve a complete gluten extraction.
• Baking changes gluten protein extractability.
• Protein extractability is decreased due to crosslinking.

## Graphical abstract

**Figure:**
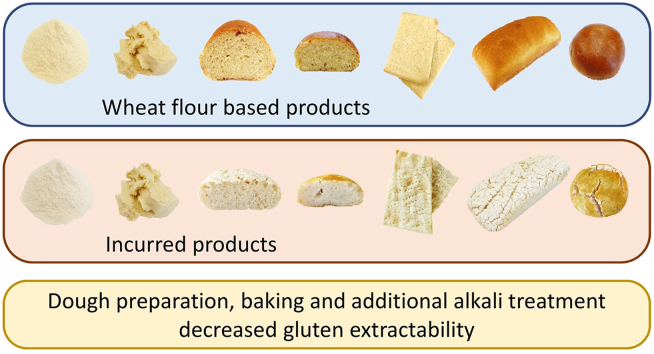

## 1 Introduction

Gluten, the storage proteins of wheat, consist of two major fractions: mostly monomeric and partially oligomeric gliadins and polymeric glutenins. Based on their relative molecular masses (M_r_) and similar amino acid sequences, gliadins are subdivided into α-, γ- and ω-gliadins, and glutenins into low-molecular-weight (LMW) and high-molecular-weight (HMW) glutenin subunits (GS) (Wieser et al., 2020, 2022). When mixed with water they form a cohesive viscoelastic network and undergo further structural changes during thermal treatment. This property of gluten makes wheat suitable for the preparation of a great diversity of staple foods such as bread or pasta. Therefore, wheat is one of the most cultivated crops worldwide (FAOSTAT, 2022). Understanding gluten network formation is important for product quality as well as for analytical gluten detection methods, since there is at least 1% of the human population who suffer from celiac disease and need to follow a gluten-free diet (Lerner et al., 2019).

Many studies that investigated gluten network formation during dough preparation and baking are based on the solubility properties of gliadins and glutenins. Gliadins are soluble in aqueous alcohols. Solvents containing denaturing agents such as sodium dodecyl sulphate (SDS) and/or urea are also often used (Delcour et al., 2012). In contrast to gliadins, which contain only intramolecular or no disulfide (SS) bonds, glutenins are connected through intermolecular SS bonds. After reduction of the SS bonds, they become soluble in aqueous alcohol similar to gliadins (Wieser et al., 2022).

Heat treatment of gluten leads to large protein aggregates with further polymerisation of glutenins, formation of gliadin-glutenin bonds and oxidation of the free thiol groups (SH) of cysteine. The incorporation of gliadins into the glutenin structure via intermolecular SS bonds results from SH-SS interchange reactions (Wieser, 1998; Lagrain et al., 2008a). Besides SS crosslinking, gluten network formation also involves non-covalent interactions (hydrogen, ionic, hydrophobic) as well as covalent non-SS crosslinking (Wieser et al., 2022). Additional non-SS bonds between gluten proteins and other food matrix components formed during thermal processing are tyrosine-tyrosine (Tilley et al., 2001), tyrosine-dehydroferulic acid (Piber and Koehler, 2005), isopeptide (Rombouts et al., 2011), Maillard (Gerrard et al., 2003; Dai et al., 2007) and dehydro amino acid derived crosslinks (Rombouts et al., 2010).

Currently, the recommended method for gluten analysis to assess whether gluten-free products test below the threshold of 20 mg/kg is the R5 ELISA using the so-called Cocktail as extraction solvent (CXS 118-1979, 2008). It contains a reducing agent (2-mercaptoethanol) and a disaggregating agent (guanidine hydrochloride) in aqueous buffer and was developed for a complete extraction of gliadins from both unprocessed and heat processed foods (García et al., 2005). Due to the toxicity and incompatibility of the Cocktail with other immunoassays (Doña et al., 2008; Mena et al., 2012), several alternative extraction solvents have been described (Gessendorfer et al., 2010; Mena et al., 2012; Ito et al., 2016; Segura et al., 2021), including universal gluten extraction solution (UGES) and universal prolamin and glutelin extractant solution (UPEX).

The effect of thermal processing on gluten detection in wheat-based products by ELISA has been investigated by Bugyi et al. (2013), Gomaa and Boye (2013), Sharma et al. (2013), Török et al. (2014) and Pahlavan et al. (2016). In general, the detectability of gluten in processed foods such as bread or cookies is negatively affected and processing conditions such as heating temperature and time are important factors. The decrease of gluten recovery after baking is explained by loss of antibody-binding activity and/or solubility of gliadin due to the altered protein structure (Bugyi et al., 2013; Török et al., 2014; Pahlavan et al., 2016). Furthermore, the sensitivity of the detection method to a specific antigen can be influenced by the extraction buffer and protocol applied (Gomaa and Boye, 2013). However, a lower detection of gluten does not necessarily indicate a decreased immunoreactivity for celiac disease or wheat allergic patients (Taylor et al., 2002; Sharma et al., 2013).

So far, studies on the influence of food processing on gluten detection by ELISA did not provide a concurrent analysis of their sample material, leaving a knowledge gap between the altered gluten structure and the detection ability.

Our aim was to contribute to a better understanding of the complex structural changes of gluten during processing leading to a decreased gluten detection. We prepared different baked goods (bread, crispbread, pretzel) and tested different extraction solvents (Cocktail, UGES, UPEX) to achieve the best possible ELISA results. This was followed by a comprehensive analysis of gluten extractability, composition and thiol concentrations to examine gluten network interconnectivity.

## 2 Material and methods

### 2.1 Material

Wheat, cultivar Akteur, harvested 2015, was kindly provided by I.G. Pflanzenzucht GmbH (Munich, Germany). The kernels were milled into flour using a Quadrumat Junior laboratory mill (Brabender, Duisburg, Germany) according to AACCI Method 26 - 50.01, sieved (mesh size 200 μm) and stored at 22 °C for 2 weeks before use. Gluten-free rice flour was donated by BFree Foods (Dublin, Ireland). Brauerei Wieninger (Teisendorf, Germany) provided fresh baker's yeast. R5 ELISA Ridascreen Gliadin (R7001) and Cocktail (patent WO 02/092633 A1) were from R-Biopharm AG (Darmstadt, Germany). UGES (patent ES 2 392 412 A1) was from Biomedal Diagnostics (Seville, Spain). PageRuler Unstained Protein Ladder and NuPage Bis-Tris Plus Gel (10%, pH 6.4, 1.0 mm × 10 wells) were from Thermo Fisher Scientific (Braunschweig, Germany). All chemicals were of analytical or higher quality and were from Merck KGaA (Darmstadt, Germany), Sigma-Aldrich (Steinheim, Germany) or SERVA Electrophoresis GmbH (Heidelberg, Germany). Prof. Dr. Koehler, Chairman of the Working Group for Prolamin Analysis and Toxicity, provided the reference material Prolamin Working Group (PWG)-gliadin (van Eckert et al., 2006). Water for chromatographic separations was purified using a Milli-Q Gradient A10 system (Millipore, Schwalbach, Germany).

### 2.2 Preparation of wheat flour-based products

*Dough*: Dough was made by mixing flour with 3% yeast and 2% NaCl using a kitchen aid (1 min, level 1) and addition of water as determined according to AACC method 54-50.01. The dough was kneaded for 3 min at level 1 and 3 min at level 3. One portion for analysis was immediately frozen and freeze-dried. The remaining dough was placed in a water-saturated proofer to rest for 20 min at 30 °C. *Crispbread*: The fermented dough was rolled as thin as possible and pricked all over with a fork before baking (9 min at 230 °C). *Bread*: About 300 g of the fermented dough was manually moulded, and put into a tin pan for proofing for 40 min (at 30 °C, 90% relative humidity) and baking (25 min at 230 °C with initial steam injection). *Pretzel*: Similar to the bread preparation, pieces of approx. 55 g of the fermented dough were hand-shaped into balls and proofed for 40 min (at 30 °C, 90% relative humidity). Afterward, the products were refrigerated (15 min, 5 °C) and shortly dipped in a 1.25 mol/L sodium hydroxide (NaOH) solution before baking (17 min at 230 °C with initial steam injection).

After baking, all baked goods were cooled for 2 h at 22 °C. Bread and pretzels were manually separated into crumb and crust. All baked goods were freeze-dried and milled (6000 rpm, 200 μm ring sieve) using an Ultra Centrifugal Mill ZM 200 (Retsch, Haan, Germany). The resulting sample material was stored air tight at 22 °C.

### 2.3 Preparation of wheat flour-incurred products

#### 2.3.1 Wheat flour-spiked rice flours

Gluten-free rice flour was spiked with wheat flour (100.7 ± 1.4 mg gluten/g as determined by RP-HPLC) to obtain three spiked rice flour mixes containing 20, 50 and 100 mg gluten/kg, respectively. The spiking was performed in two steps. The rice flour was mixed with wheat flour to obtain a dilution of 1000 mg gluten/kg. This mixture was used to prepare the final dilutions. All spiked samples were mixed in a Turbula shaker-mixer (Glen Mills Inc., Clifton, USA) for 12 h to ensure sufficient homogeneity, as reported earlier (Schopf and Scherf, 2018).

#### 2.3.2 Preparation of incurred baked goods

Crispbread, bread and pretzels were produced from each of the three spiked rice flour mixes (20, 50, 100 mg gluten/kg) as described above. The addition of water was 64% (w/w).

### 2.4 Crude protein and moisture content

The nitrogen content of wheat flour and wheat flour-based products was determined according to the method of Dumas using a TruSpec Nitrogen Analyzer (Leco, Moenchengladbach, Germany) and converted to crude protein content using a conversion factor of 5.7 as stated in ICC Standard 167 (International Association for Cereal Science and Technology, 2000). The moisture content of all flours and flour-based products was determined using an Infrared Moisture Analyzer MA35 (Sartorius, Goettingen, Germany) at 100 °C until the residual weight remained constant. All measurements were performed in triplicates.

### 2.5 R5 ELISA

The R5 ELISA Ridascreen Gliadin was performed according to the manufacturer's instructions. Wheat flour, wheat flour-based, wheat flour-spiked and wheat flour-incurred samples (3 replicates for samples containing 20 mg gluten/kg, 6 replicates for all other samples) were extracted with either 2.5 mL Cocktail, UGES or UPEX. Cocktail and UGES were purchased. UPEX (5 mmol/L tris(2-carboxyethyl)phosphine (TCEP), 2% (w/v) *N*-lauroylsarcosine in phosphate buffered saline (PBS), pH 7.0) was prepared according to Mena et al. (2012). TCEP and *N*-lauroylsarcosine were added to the PBS immediately before use. Samples were diluted with 60% (v/v) ethanol and in the last dilution step with the particular extraction solvent to final dilution factors of 500 for 20 mg gluten/kg of spiked flour and incurred materials, of 1000 for 50 mg gluten/kg of spiked flour and incurred materials, of 2500 for 100 mg gluten/kg of spiked flour and incurred materials, and of 2,500,000 for wheat flour and wheat flour-based products. The cubic spline function of the software RIDASOFT Win (version 1.93) was used to calculate the gliadin content from the absorbance at 450 nm. The dry matter of the sample material was considered.

### 2.6 SDS-PAGE

SDS-PAGE was carried out according to Lagrain et al. (2012) using a XCELL Surelock Mini-Cell electrophoresis system (Thermo Fisher Scientific, Braunschweig, Germany), a homogeneous NuPAGE Bis-Tris gel (10%) and a PageRuler Unstaind Protein Ladder as molecular weight marker. In brief, about 25 mg of wheat flour and wheat flour-based products were dissolved in 1 mL of extraction buffer and incubated under reducing conditions overnight at 22 °C. Then, the suspensions were shaken for 10 min at 700 rpm and 60 °C and centrifuged. The supernatants (10 μL) were loaded onto the gel. Gel electrophoresis was performed with a 3-(*N*-morpholino)propanesulfonic acid (MOPS) running buffer and the following running conditions: current: 115 mA; voltage 200 V, power: 30 W, time: 20 - 30 min. Proteins were fixed for 30 min with 12% trichloroacetic acid, stained for 30 min with 0.25% Coomassie Brilliant Blue R-250 and destained for 15 min with methanol/water/acetic acid (50/40/10, v/v/v) and in water/methanol/acetic acid (80/10/10, v/v/v) overnight.

### 2.7 Determination of protein composition by Osborne fractionation

Modified Osborne fractionation was carried out according to Wieser et al. (1998). All samples were extracted in triplicate. In brief, wheat flour and wheat flour-based products (100 mg) were extracted twice with 1.0 mL 0.067 mol/L Na_2_HPO_4_/KH_2_PO_4_ buffer (pH 7.6) and 0.4 mol/L NaCl (salt-soluble proteins). Then, the residues were extracted three times with 0.5 mL 60% (v/v) ethanol (alcohol-soluble proteins). Last, the residues were extracted twice with 1.0 mL of 50% (v/v) propan-1-ol, 0.05 mol/L Na_2_HPO_4_/KH_2_PO_4_ (pH 7.5) and 1% (w/v) DTT under nitrogen atmosphere at 60 °C (alcohol-insoluble proteins).

An UltiMate 3000 instrument (Dionex, Idstein, Germany) with an Acclaim 300 C_18_ column (2.1 × 150 mm, Thermo Fisher Scientific, Braunschweig, Germany) and the software Chromeleon was used for reversed-phase high-performance liquid chromatography (RP-HPLC). The instrument was set to: column temperature: 60 °C, flow rate: 0.2 ml/min, injection volume: 20 - 50 μL (salt-soluble proteins), 10 - 20 μL (alcohol-soluble and alcohol-insoluble proteins), solvent: water/trifluoroacetic acid (TFA) (999/1, v/v) (A) and acetonitrile (ACN)/TFA (999/1, v/v) (B), gradient for salt-soluble proteins: 0 min 0% B, 0.5 min 20% B, 7 min 60% B, 7.1 min 90% B, 11.1 min 0% B, gradient for alcohol-soluble and alcohol-insoluble proteins: 0 min 0% B, 0.5 min 24% B, 20 min 56% B, 20.1 min 90% B, 24.2 min 0% B, detection: UV absorbance at 210 nm. PWG-gliadin in 60% (v/v) ethanol was used for an external calibration. The peaks for salt-soluble proteins had a retention time of 5.0 - 12.0 min. The peak profiles of alcohol-soluble and alcohol-insoluble protein extracts were further divided into four or three fractions, respectively, as described by Wieser (1998). ω5-Gliadins occurred from 7.0 to 10.8 min, ω1,2-gliadins from 10.8 to 13.2 min, α-gliadins from 13.2 to 16.5 min and γ-gliadins from 16.5 to 22.0 min. Glutenin-bound ω-gliadins (ωb-gliadins) were eluted from 7.0 to 10.8 min, HMW-GS from 10.8 to 14.0 min and LMW-GS from 14.0 to 22.0 min. Protein content is given as mg/g based on dry matter.

### 2.8 Determination of SDS-soluble proteins and SDS-insoluble proteins

The extraction protocol was adapted from Thanhaeuser et al. (2014). All samples were extracted in triplicate. Wheat flour and wheat flour-based products (100 mg) were extracted twice with 1.0 mL of 0.05 mol/L Na_2_HPO_4_/KH_2_PO_4_ buffer (pH 6.9) and 1% (w/v) SDS (SDS-soluble proteins). Then, the residues were extracted twice again with 1.0 mL of 50% (v/v) propan-1-ol, 0.05 mol/L Na_2_HPO_4_/KH_2_PO_4_ buffer (pH 7.5) and 1% (w/v) DTT under nitrogen atmosphere at 60 °C (SDS-insoluble proteins). The suspensions were centrifuged (20 min, 4600×*g*, 22 °C) and the resulting supernatants were combined. Supernatants of the first extraction were filled up to 5.0 mL, supernatants of the second extraction up to 2.0 mL with the corresponding extraction solution. All supernatants were filtered (Whatman, Spartan 13/0.45 RC, GE Healthcare, Freiburg, Germany) prior to analysis by gel permeation(GP)-HPLC.

An XLC instrument (Jasco, Gross-Umstadt, Germany) with a BioSep SEC S3000 column (4.6 × 300 mm, Phenomenex, Aschaffenburg, Germany) and the software ChromPass was used for the analysis of the protein fractions. The instrument was set to: column temperature: 22 °C, flow rate: 0.3 ml/min, injection volume: 20 - 60 μL (SDS-soluble proteins), 5 - 10 μL (SDS-insoluble proteins), solvent: water /TFA (999/1, v/v) (A) and ACN/TFA (999/1, v/v) (B), isocratic 50% A/50% B, detection: UV absorbance at 210 nm.

PWG-gliadin was used for an external calibration as described above. The peaks had a retention time of 6.0 - 13.0 min. The peak profiles of SDS-insoluble protein extracts were further divided into three fractions as described by Thanhaeuser (2015). HMW-GS appeared from 6.0 to 8.2 min, LMW-GS from 8.2 to 10.3 min and residual ALGL from 10.3 to 13.0 min. Protein contents are given as mg/g based on dry matter.

### 2.9 Determination of alcohol-soluble proteins by GP-HPLC

Wheat flour and wheat flour-based products (100 mg) were extracted twice directly with 1.0 mL of 60% (v/v) ethanol as described above. After splitting into two aliquots, one aliquot was further incubated under reducing conditions with excess DTT for 30 min at 60 °C. GP-HPLC analysis was performed with an Extrema LC-4000 instrument (Jasco, Gross-Umstadt, Germany) as described above. Retention times were assigned to relative M_r_ ranges by analysing a gel filtration M_r_ marker kit from Sigma-Aldrich (Steinheim, Germany). It contained the following proteins: β-amylase (200 kDa), alcohol dehydrogenase (150 kDa), albumin (66 kDa), carbonic anhydrase (29 kDa) and cytochrome C (12.4 kDa). The M_r_ markers of 66 and 200 kDa eluted in one peak, to which proteins with M_r_ ranging from 60 to 200 kDa were assigned. All samples were extracted in triplicate.

### 2.10 Determination of free thiols and disulfide bonds

Wheat flour and wheat flour-based products (10 mg) were suspended in 900 μL of Na_2_HPO_4_/NaH_2_PO_4_ buffer (0.05 mol/L, pH 6.5, 3 mol/L urea, 0.001 mol/L EDTA and 2% (w/v) SDS), further referred to as sample buffer. The samples were shaken for 60 min at 500 rpm and 22 °C. Then, 100 μL of 5,5′-disulfanediylbis(2-nitrobenzoic acid) (0.1% (w/v) in sample buffer) was added and the mixtures were incubated for 45 min at 500 rpm and 22 °C. Samples were centrifuged (11,000×*g*, 5 min, 20 °C). The absorbance of the supernatants was immediately measured with an UV-2401PC spectrophotometer (Shimadzu, Kyoto, Japan) at 412 nm. Controls containing no sample were used to correct for background absorbance. Absorbance values were converted to levels of free SH (μmol SH/g protein) using a calibration with reduced glutathione (0 - 15 μmol). To quantitate SS bonds, samples were reduced with 200 μL of NaBH_4_ (2.5%, w/v) and shaken for 60 min at 500 rpm and 50 °C. Excess NaBH_4_ was degraded by adding 100 μL HCl (1 mol/L) and further shaking for 30 min at 500 rpm and 22 °C. Free SH were determined as described earlier. Oxidized glutathione was used for calibration (0 - 15 μmol). Levels of SS bonds (μmol SS/g protein) were corrected for the amount of free SH (subtraction of mean).

### 2.11 Determination of cysteine and glutathione

The concentrations of total glutathione (GSH) and cysteine (CSH) were determined by a stable isotope dilution assay using LC-MS/MS as described by Schmid et al. (2016). For the reduction of SS bonds, 20 mg of wheat flour and wheat flour-based processed foods were suspended in 200 μL of an aqueous TCEP solution (5 mg/mL). Then, 20 μL of internal standard GSH ([^13^C_2_], [^15^N]; c = 25 μg/mL) and CSH ([^13^C_3_], [^15^N]; c = 16 μg/mL) dissolved in 5% (v/v) perchloric acid, as well as 200 μL TCEP (c = 5 mg/mL) were added. Samples were shaken for 30 min at 500 rpm and 22 °C in the dark. Then, 600 μL of iodoacetic acid (0.02 mol/L) in a H_3_BO_3_/LiOH buffer (0.5 mol/L, pH 8.5) were added. Samples were shaken for 30 min at 500 rpm and 22 °C in the dark. Afterward, 500 μL dansyl chloride (3.7 mmol/L in ACN) was added, and the mixture was shaken again for 1 h at 500 rpm and 22 °C in the dark. Last, 500 μL of dichloromethane was added, quickly mixed using a vortex mixer and centrifuged (16,000×*g*, 10 min, 22 °C). The supernatants were filtered and centrifuged in Vivaspin centrifugal concentrators (cut-off <3000 g/mol, 16,000×*g*, 22 °C) overnight. The permeates were analysed using a TSQ Quantum Discovery LC-MS/MS system coupled with a Finnigan Surveyor Plus HPLC system (Thermo Fisher, Braunschweig, Germany) with a Synergi Hydro-RP column (2 × 150 mm, 4 μm particle size, 8 nm pores, Phenomenex, Germany) and the software Skyline (version 4.2.0). The HPLC system was set to: flow rate: 0.2 ml/min, injection volume: 10 μL, solvent: water/formic acid (FA) (999/1, v/v) (A) and ACN/FA (999/1, v/v) (B), linear gradient: 0 min 0% B, 25 min 100% B. The MS was operated in electrospray ionization positive mode with capillary temperature: 290 °C, sheath gas pressure: 30 arbitrary units, auxiliary gas pressure: 10 arbitrary units, spray voltage: 4 kV, capillary offset: 35 V, collision cell pressure: 67 Pa, scan time 200 ms.

### 2.12 Statistical analysis

One-way analysis of variance (one-way ANOVA) with Tukey's test at a significance level of p < 0.05 was applied to determine significant differences between analysis results (SigmaPlot version 12.0, Systat Software, San Jose, USA).

## 3 Results

### 3.1 Investigation of different extraction solvents for ELISA

**Fig. 1:**
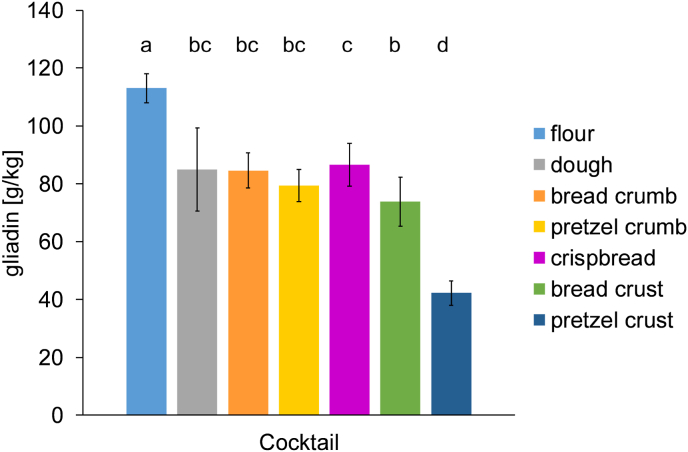
Gliadin content of wheat flour, dough and baked goods quantitated by R5 ELISA after extraction with Cocktail. Data represented are the means ± standard deviation (n = 3). Means with different small letters are significantly different (one-way ANOVA, Tukey's test, р ≤ 0.05).

The wheat flour and wheat flour-based samples were extracted only with Cocktail since gluten analysis by ELISA is primarily relevant for low gluten products. Fig. 1 shows that the gliadin content of bread, crispbread and pretzel samples was significantly lower than the gliadin content of the wheat flour. Pretzel crust had the lowest detectable gliadin content (42 g/kg).

**Fig. 2:**
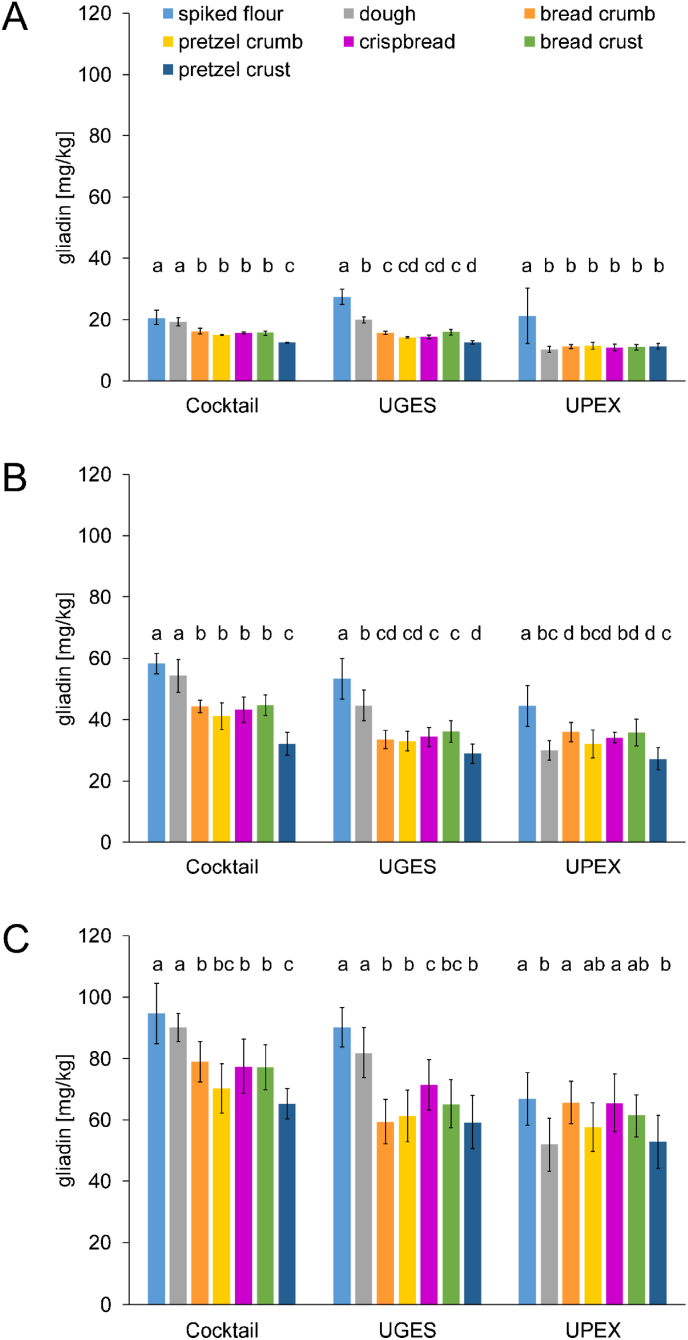
Gliadin content of wheat flour-spiked rice flour, dough and baked goods quantitated by R5 ELISA after extraction with Cocktail, UGES (universal gluten extraction solution) and UPEX (universal prolamin and glutelin extractant solution). Target levels of 20 mg gluten/kg (A), 50 mg gluten/kg (B) and 100 mg gluten/kg (C). Data represented are the means ± standard deviation (n = 3). Different small letters represent a significant difference (one-way ANOVA, Tukey's test, р ≤ 0.05) between samples extracted with the same extraction solvent.

Samples, which were spiked or incurred with different levels of wheat flour (20, 50, 100 mg gluten/kg) were additionally extracted with UGES and UPEX (Fig. 2). The gliadin recovery is expressed as gliadin content analysed relative to the spiked gluten protein content in the following. Alternatively, the gliadin content could be multiplied by 2, which is recommended as gliadin to gluten conversation factor by the Codex Alimentarius (CXS 118-1979, 2008). However, studies have shown that this factor is inaccurate in many cases leading either to over- or underestimation of the real gluten content (Wieser and Koehler, 2009).

All extraction solvents provided a good gliadin recovery (>80%) for the flours spiked with 20 and 50 mg gluten/kg. Sufficient gliadin was also extracted with Cocktail and UGES from their corresponding doughs. The gliadin content of all baked goods incurred with 20 and 50 mg gluten/kg was significantly lower than that of the spiked flours (Fig. 2 AB). No extraction solvent could compensate the effect of baking on the detection. UPEX showed the lowest extraction efficiencies, especially for the baked goods incurred with 20 mg gluten/kg, but also the dough samples. Results for samples spiked and incurred with 100 mg/kg gluten were similar to those with 20 and 50 mg gluten/kg (Fig. 2 C). However, the extractability of gliadin from flour spiked with 100 mg gluten/kg was less with UPEX in comparison to the other extraction solvents (67 mg/kg in comparison to > 90 mg/kg). For this reason, effects of the baking process were less significant if extracted with UPEX. In general, gliadin levels of bread, crispbread and pretzel samples ranged from about 16–45% less in Cocktail extracts, about 21–54% less in UGES extracts and about 2–48% less in UPEX extracts in comparison to their corresponding flours. The extraction with Cocktail achieved a slightly better gliadin recovery from baked goods in comparison to the extraction with UGES and UPEX. There was no clear correlation between the gliadin content analysed and the degree of heating, because the gliadin contents analysed in the crumb, crispbread, and bread crust were in the same range. However, additional alkali treatment of the pretzel crust resulted in the lowest gliadin content as also seen for the wheat flour-based samples (<12 mg/kg in 20 mg gluten/kg, <33 mg/kg in 50 mg gluten/kg, <66 mg/kg in 100 mg gluten/kg).

### 3.2 Investigation of protein extractability

#### 3.2.1 Qualitative protein composition

**Fig. 3:**
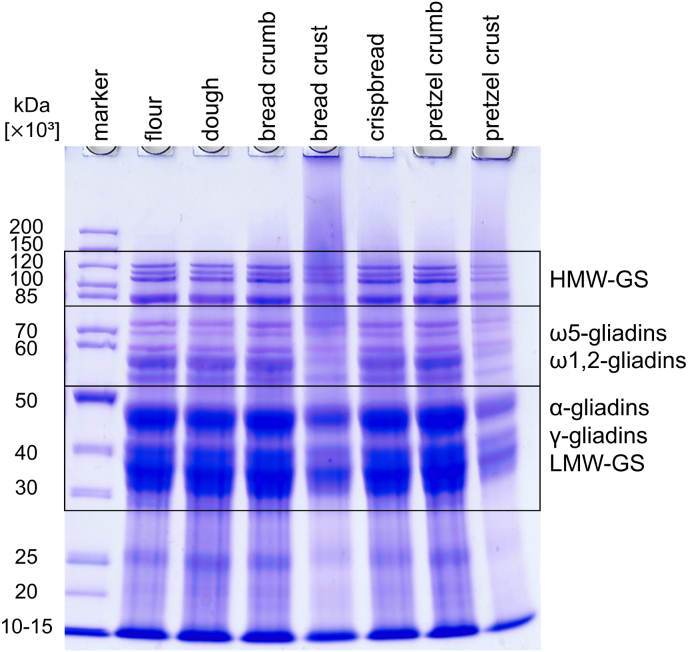
SDS-PAGE (10% Bis-Tris) of total protein extracts from wheat flour, dough and baked goods. HMW-GS, high-molecular-weight glutenin subunits; LMW-GS, low-molecular-weight glutenin subunits.

SDS-PAGE of protein extracts from wheat flour, dough, bread, crispbread and pretzel samples showed the expected band patterns typical for wheat proteins, cv. Akteur, as described by Lagrain et al. (2012) (Fig. 3). The band patterns of all samples were similar, indicating that the qualitative composition was comparable. Only the bread crust and pretzel crust showed a weaker intensity of some bands, especially bands related to α- and γ-gliadins and LMW-GS. Both crust samples also contained very large protein aggregates, which did not migrate into the gel.

#### 3.2.2 Quantitative protein composition

**Fig. 4:**
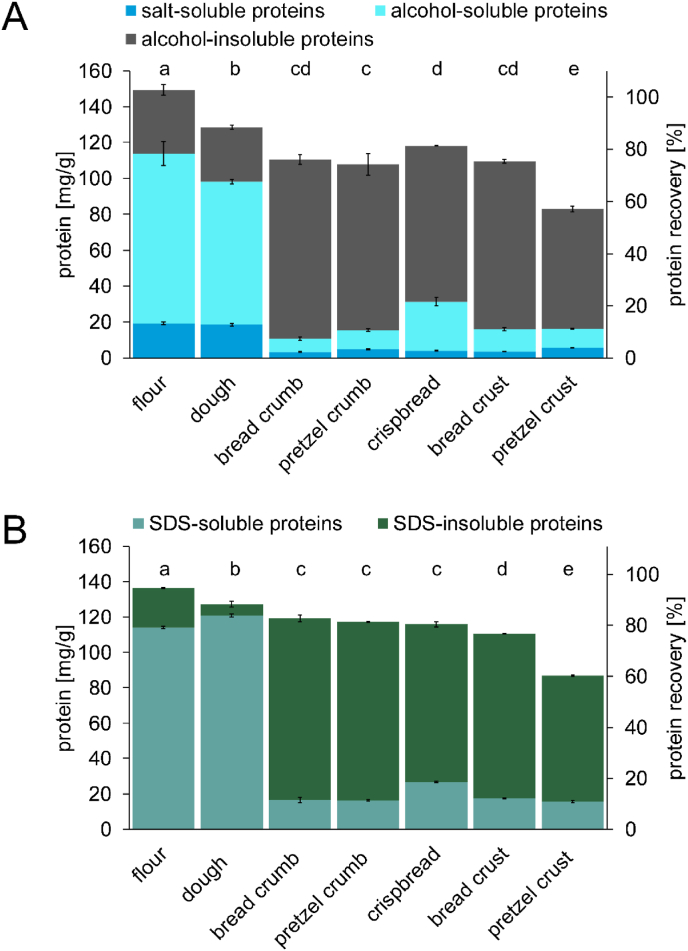
Concentrations of salt-soluble, alcohol-soluble and alcohol-insoluble proteins determined by RP-HPLC (A), SDS-soluble and SDS-insoluble proteins determined by GP-HPLC (B), and protein recovery calculated as percentage of the extracted proteins relative to the total crude protein content. Data represented are the means ± standard deviation (n = 3). Means of protein recovery with different small letters are significantly different (one-way ANOVA, Tukey's test, р ≤ 0.05).

The quantitative protein composition of the wheat flour, dough, bread, crispbread and pretzel samples was determined by RP-HPLC and by GP-HPLC (Fig. 4). Independent of the extraction protocol and the kind of HPLC analysis, dough preparation and baking decreased protein recovery, which is the protein content analysed relative to the total crude protein content. Baking (75 - 83% recovery) decreased protein extractability more than dough preparation (about 90% recovery). Additional alkali treatment further reduced protein recovery in the pretzel crust (58 - 61% recovery).

The proportion of salt-soluble, alcohol-soluble and alcohol-insoluble proteins of flour and dough was similar (Fig. 4 A). In contrast, baking decreased the levels of salt-soluble and alcohol-soluble proteins and increased the levels of alcohol-insoluble proteins. The composition of proteins extracted from the crumb and crust samples was similar ranging from 3.3 (bread crumb) to 5.5 (pretzel crust) mg salt-soluble protein/g, 7.4 (bread crumb) to 12.5 (bread crust) mg alcohol-soluble protein/g, and 86.8 (crispbread) to 99.8 (bread crumb) mg alcohol-insoluble protein/g. The crispbread showed a higher concentration of alcohol-soluble proteins (27.4 mg/g) than the other baked goods. The pretzel crust had the lowest protein recovery (58%), resulting in a lower content of alcohol-insoluble proteins (66.9 mg/g) in comparison to the other baked goods.

In general, the quantitation of SDS-soluble and SDS-insoluble proteins corresponded with the results of the modified Osborne fractionation. Protein recovery significantly decreased from 95% for flour to 61% for pretzel crust (Fig. 4 B). Dough preparation significantly increased the protein extractability in SDS buffer from 114.0 mg SDS-soluble protein/g flour to 120.8 mg SDS-soluble protein/g dough. As seen for salt-soluble, alcohol-soluble and alcohol-insoluble proteins, baking drastically changed the protein composition in favour of SDS-insoluble proteins. Levels of SDS-soluble proteins decreased by at least 72%. The amount of SDS-soluble and SDS-insoluble proteins extracted from the crumb and crust samples was similar ranging from 15.6 mg (pretzel crust) to 17.6 (bread crust) mg SDS-soluble protein/g and 71.3 mg (pretzel crust) to 102.6 mg (bread crumb) SDS-insoluble protein/g. Again, the crispbread showed a higher concentration of SDS-soluble proteins (26.8 mg/g). The pretzel crust had a lower content of SDS-insoluble proteins in comparison to the other baked samples due to the low protein recovery (61%).

**Fig. 5:**
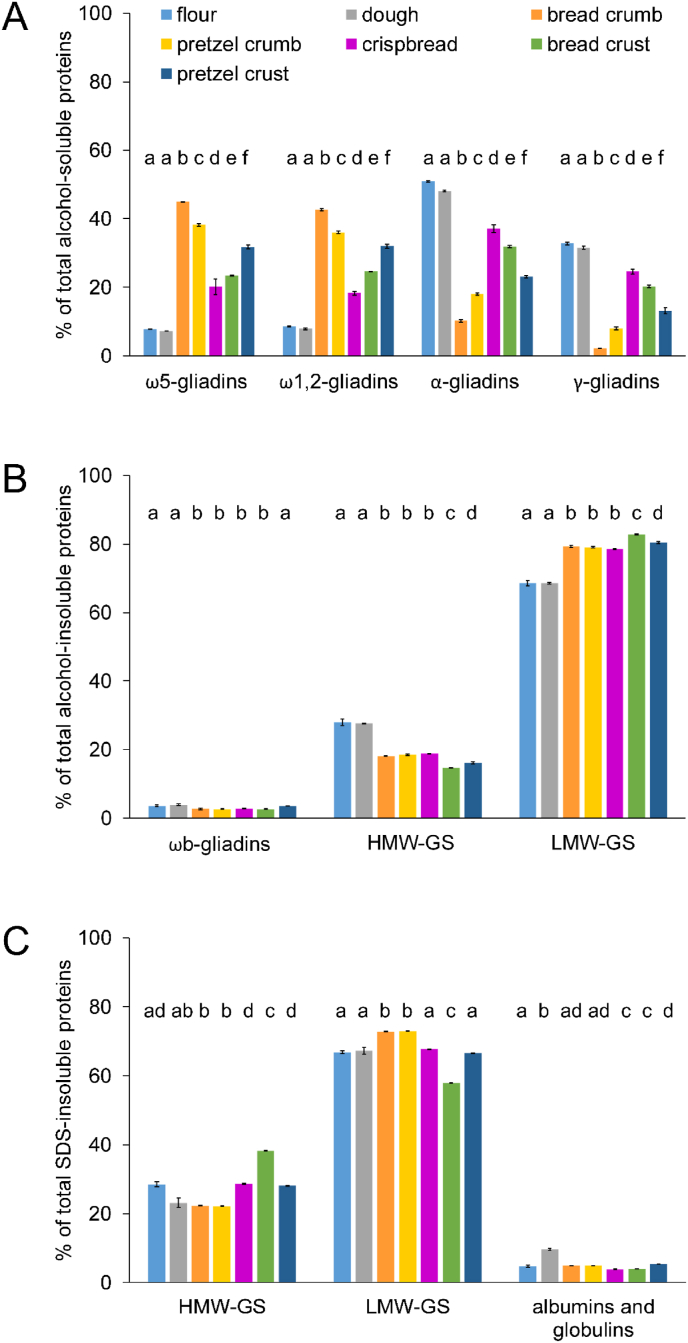
Relative concentrations of ω5-, ω1,2-, α- and γ-gliadins from extracted alcohol-soluble proteins (A) and of ωb-gliadins, high-molecular-weight (HMW) and low-molecular-weight (LMW) glutenin subunits (GS) from extracted alcohol-insoluble proteins determined by RP-HPLC (B), as well as HMW-GS, LMW-GS, albumins and globulins from extracted SDS-insoluble proteins determined by GP-HPLC (C). Data represented are the means ± standard deviation (n = 3). Different small letters represent a significant difference (one-way ANOVA, Tukey's test, р ≤ 0.05) between samples within one gluten protein type.

The alcohol-soluble, alcohol-insoluble and SDS-insoluble protein fractions were further analysed as described above. The alcohol-soluble protein fraction was subdivided into ω5-, ω1,2-, α- and γ-gliadins (Fig. 5 A). The alcohol-insoluble protein fraction was subdivided into ω_b_-gliadins, HMW-GS and LMW-GS, and the SDS-insoluble fraction into HMW-GS and LMW-GS as well as residual albumins and globulins (Fig. 5 BC). Flour and dough did not significantly differ within the gliadin types. In comparison to flour and dough (8% ω5-gliadins, 9% ω1,2-gliadins, 51% α-gliadins, 33% γ-gliadins), the baked goods had higher proportions of ω5-and ω1,2-gliadins with values of about 20% (crispbread) to 45% (bread crumb) and lower proportions of α- and γ-gliadins with values of about 10% (bread crumb) to 38% (bread crust) for α-gliadins, and 2% (bread crumb) to 25% (crispbread) for γ-gliadins. The ω_b_-gliadins showed the lowest proportions overall (<4%), further reduced in the bread crumb, bread crust and crispbread.

The alcohol-insoluble HMW-GS and LMW-GS showed the same proportions for flour and dough (28% HMW-GS, 69% LMW-GS). In comparison to the flour, the relative proportions of HMW-GS in the baked goods were lower, ranging from 15% (bread crust) to 19% (crispbread) and higher for LMW-GS, ranging from 79% (crispbread) to 83% (bread crust).

In general, there was no clear effect of the additional alkali treatment (pretzel crust) on the composition of gluten protein types. The absolute levels of all alcohol-soluble gliadins decreased in the bread, crispbread and pretzel samples; α- and γ-gliadins to a greater extent than ω-gliadins. Concurrently, absolute levels of all types of alcohol-insoluble proteins were increased by the baking process. The quantitation of HMW-GS and LMW-GS of the SDS-insoluble protein fraction from flour, dough and crumb samples were in accordance with the results for the alcohol-insoluble protein fraction. However, there was no clear trend of HMW-GS, LMW-GS as well as the residual albumin and globulin proportions within the samples of the SDS-insoluble protein fraction.

**Fig. 6:**
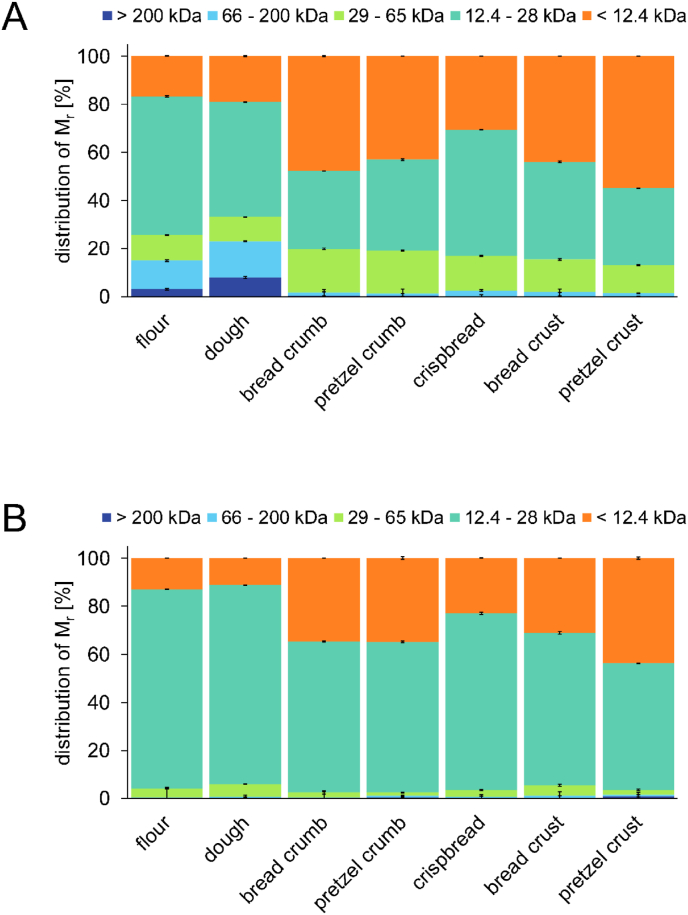
Relative molecular mass (M_r_) distribution of alcohol-soluble proteins (A) and alcohol-soluble proteins after reduction with DTT (B) determined by GP-HPLC. Data represented are the means ± standard deviation (n = 3).

To further investigate changes of the gliadin composition caused by bread, crispbread and pretzel making, an additional extraction of all alcohol-soluble proteins (including salt-soluble proteins) was performed and the proteins were analysed regarding their M_r_ distribution before and after reduction with DTT. It revealed that proteins with M_r_ of >66 kDa (15 - 23%) became alcohol-insoluble after the baking process (Fig. 6 A). These proteins could be reduced almost completely if treated with DTT (Fig. 6 B). The alcohol-soluble proteins extracted from bread, crispbread and pretzel samples showed higher proportions of proteins with M_r_ of <12.4 kDa and 29 - 66 kDa, and a lower proportion of proteins with a M_r_ of 12.4 - 29 kDa in comparison to the flour and dough. Most proteins with a M_r_ of 29 - 66 kDa could be reduced. After reduction, the crispbread had the highest proportion of proteins with a M_r_ of 12.4 - 29 kDa (74%) and the lowest proportion of very small proteins with a M_r_ of <12.4 kDa (23%) of all baked goods, thus were closest to the M_r_ distribution of flour in general. The opposite was true for the pretzel crust.

### 3.3 Content of S-containing groups and compounds

**Fig. 7:**
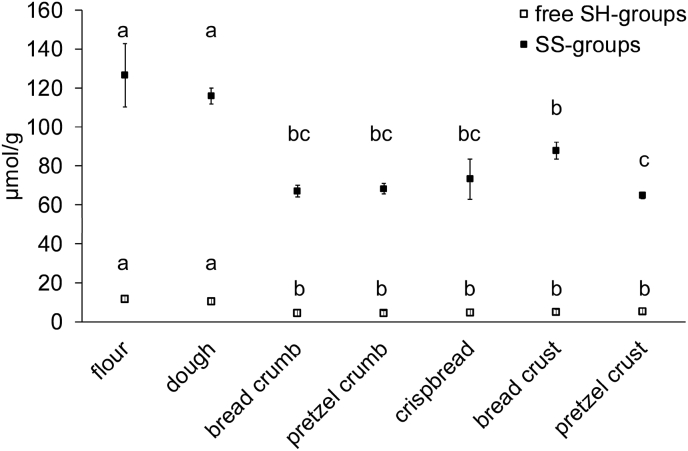
Concentrations of disulfide (SS) and free thiol (SH) groups determined spectrophotometrically. Data represented are the means ± standard deviation (n = 3). Means with different small letters are significantly different (one-way ANOVA, Tukey's test, р ≤ 0.05).

The content of free SH and SS bonds was analysed using the Ellmann method (Fig. 7). In bread, crispbread and pretzel samples both SH and SS bonds decreased from about 11 μmol SH/g (flour, dough) to about 5 μmol SH/g and from about 120 μmol SS/g (flour, dough) to less than 88 μmol SS/g. The content of free SH did not differ between the samples of baked goods. However, the crispbread had a higher content of SS bonds (87.8 μmol SS/g) than the other baked goods.

**Fig. 8:**
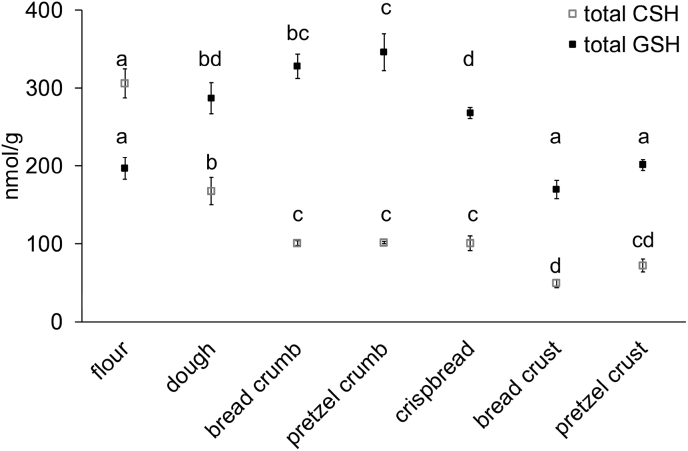
Concentrations of total cysteine (CSH) and total glutathione (GSH) determined by stable isotope dilution assay LC-MS/MS. Data represented are the means ± standard deviation (n = 3). Means with different small letters are significantly different (one-way ANOVA, Tukey's test, р ≤ 0.05).

The content of total CSH and total GSH was determined by SIDA LC-MS/MS. Fig. 8 shows that the total CSH content decreased with the degree of baking from 306 nmol CSH/g in the flour to <72 nmol CSH/g in the bread crust and pretzel crust. After the addition of yeast, the content of total GSH in the dough (287 nmol GSH/g), bread crumb (328 nmol GSH/g), pretzel crumb (345 nmol GSH/g) and crispbread (268 nmol GSH/g) was higher than in the flour (197 nmol GSH/g). It is likely that the sample preparation with perchloric acid induced a release of GSH from yeast cells (Hahn, 1996). The total GSH concentrations of the flour and crust samples were the same, which hence corresponded to an actual decrease in the crust samples. Within the baked goods, there was no further effect of alkali treatment on the concentration on S-containing groups and compounds in the pretzel crust.

## 4 Discussion

We used different baked goods (bread, crispbread, pretzel) made from one dough as model system to reflect different degrees of processing. The bread and pretzel were separated into crumb and crust. These components had been exposed to different processing conditions, that is the baking temperature (Zanoni et al., 1993; Ogilvie et al., 2021), steam injection and alkaline dipping, favouring Maillard and caramelization reactions in the crust samples (Dessev et al., 2020). The crispbread baking conditions varied by a shorter baking time and no steam injection. According to an increasing degree of processing the sample material was ranked as follows: flour, dough, bread crumb, pretzel crumb, crispbread, bread crust and pretzel crust. To cover the legislative threshold levels of gluten-free and very low gluten foods as specified in the European Commission Regulation (EC) No 41/2009, also gluten-reduced versions (20, 50, 100 mg gluten/kg) were prepared.

A commercial sandwich R5 ELISA was used to determine the gliadin content after extraction with Cocktail, UGES and UPEX. The extraction solutions contained different disaggregating and reducing agents. Disaggregating agents used were the chaotropic guanidine hydrochloride (Cocktail), the amino acid arginine (UGES) and the detergent N-lauroylsarcosine (UPEX). Reducing agents used were 2-mercaptoethanol (Cocktail) and TCEP (UPEX). The reducing agent contained in UGES is not disclosed yet (Segura et al., 2021). The mode of action of the disaggregating agents as well as the strength of the reducing agents is different, which can explain the discrepancies of the extraction efficiency. The precise mechanism and interplay of the different agents in the context of gluten solubilisation needs further clarification, though.

In general, all extraction solvents achieved gliadin recovery rates, which were in the tolerated range of 80% - 120% (Abbott et al., 2010), for the flours spiked with 20 and 50 mg gluten/kg. In the extraction of flours spiked with 100 mg gluten/kg UPEX was the only extraction solvent which stayed below 80% (58%). This is only partly in agreement with previous studies (Mena et al., 2012; Segura et al., 2021), that found a good accordance in the gluten content of (gliadin spiked) commercial foods analysed by R5 sandwich ELISA in combination with Cocktail, UGES, but also UPEX extraction protocols. However, the effect of processing on gluten quantitation has only been evaluated by means of spiked materials, although incurred materials are recommended for better commutability to actual food samples. We showed that less gliadin was detected in the incurred bread, crispbread and pretzel samples in comparison to their spiked flour samples regardless of the extraction solvent. Additional alkali treatment of the pretzel crust impaired the gliadin recovery even more. Overall, the extraction with Cocktail resulted in slightly but often significantly higher recovery rates than the extraction with UGES and particularly UPEX. Furthermore, only the use of Cocktail enabled a gliadin detection in dough comparable to flour. Thus, the extraction with Cocktail seems to be the method of choice, but also faces challenges to compensate for effects of processing like baking and alkaline pH.

In our study, the conversion factor of 2, which is usually applied to calculate the gluten content based on the gliadin content analysed by ELISA, would be sufficient for a correct labelling of most samples regardless of the kind of extraction solvent or degree of processing. Three samples (flour spiked with 50 mg gluten/kg if extracted with Cocktail or UGES; dough incurred with 50 mg gluten/kg if extracted with Cocktail) would be even unnecessarily labelled gluten-containing. Thus, the conversion factor is often also a kind of safety factor, which was not originally intended. However, since this factor needs to cover highly variable conditions such as different gliadin/glutenin ratios depending on the type of grain and raw material (flour, starch), or the degree of processing, it is very likely that there are some especially low gluten foods which still suffer from incorrect labelling. Additional data have to be generated in further studies to assess if a recovery correction could help to improve food safety in this respect. At this point, it does not seem possible to advocate for recovery correction, as long as recovery may be very different depending on the sample.

To better understand if gluten structure or gluten amount was responsible for the reduced detectability, further experiments were performed to analyse gluten composition and extractability. SDS-PAGE revealed no clear differences in the qualitative protein composition. The protein extracts of the bread crust and pretzel crust resulted in weaker bands in comparison to the other samples indicating a reduced protein extractability.

From the results of both RP- and GP-HPLC, it became clear that dough preparation, baking and additional alkali treatment of the pretzel crust decreased protein recovery under reducing and non-reducing conditions. The magnitude of extractability losses was dependent on the processing conditions with dough preparation resulting in a higher protein recovery than baking, and alkali dipping prior to baking resulting in the lowest protein recovery. Alkaline treatment further reduced the extractability of alcohol- and SDS-insoluble proteins (but not salt-, alcohol- and SDS-soluble proteins) remaining in the extraction sediment.

Baking strongly influenced protein extractability and resulted in a large decrease of salt-, alcohol- and SDS-soluble proteins, whereas dough preparation significantly increased the extractability of SDS-soluble proteins. It is still under debate whether this is due to depolymerisation, conformational rearrangement or better dissolution by a changed effective surface area of SDS-soluble proteins (Eckert et al., 1993; Weegels et al., 1997; Belton, 2005).

A better SDS-solubility of proteins, mainly of gliadins, could also explain why the gliadin content analysed by ELISA with the Cocktail extraction was similar for the spiked flours and incurred doughs, though their overall protein extractabilities were reduced. Nevertheless, this was not true for the extraction with UGES and UPEX, where the reduction of the total protein extractability also resulted in a reduced ELISA detection. Thus, the kind of disaggregating agent might play a role in ELISA, but it is difficult to compare since none of the ELISA extraction solvents uses SDS as disaggregating agent.

The reduction of SS bonds with DTT rendered the major proportion of proteins extractable in aqueous propan-1-ol. This indicated that salt-, alcohol-, and SDS-soluble proteins, which contain the major part of gliadins, became extractable with the alcohol-insoluble and SDS-insoluble protein fractions, which mainly comprises glutenins. It is known that this is due to gluten polymerisation by oxidation of the free SH groups of cysteine and/or SH-SS exchange reactions, which are favoured by mechanical and thermal treatment (Schofield et al., 1983; Kieffer et al., 2007; Lagrain et al., 2008b).

The relative accumulation of ω-gliadins, accompanied by a relative decrease of α- and γ-gliadins in our study supported the hypothesis that α- and γ-gliadins are the main drivers in gliadin-glutenin crosslinking during baking (Schofield et al., 1983; Weegels and Hamer, 1998; Lagrain et al., 2007, 2008a). α-Gliadins have three and γ-gliadins four intramolecular SS bonds, which can be incorporated via SS crosslinking into the alcohol- and SDS-insoluble glutenin fraction (Shewry et al., 1986; Shewry and Tatham, 1997). ω-Gliadins lack cysteine residues and therefore play a minor role.

A proportion of around 15 - 23% of the alcohol-soluble proteins consisted of oligomeric proteins with M_r_ of >66 kDa, known as HMW-gliadins (Wieser et al., 2020). They were completely involved in gluten polymerisation via SS crosslinking induced by the baking process.

The absolute levels of all glutenins increased, whereas only the relative proportion of LMW-GS increased. Thus SH-SS exchange of α- and γ-gliadins resulted in a shift of solubility and consequently detection together with LMW-GS. However, this could only be partially confirmed by the analysis of protein types of the SDS-insoluble protein fraction.

Of all baked goods, the crispbread most closely resembled the flour regarding the protein type composition. Since there was no clear correlation between the protein type composition and the processing conditions of the baked goods, the protein composition of the pretzel crust did not provide an explanation for the lowest detectability by ELISA.

Even the use of the strong reducing agent DTT did not restore the original protein extractability as in the flour, suggesting the contribution of not only SS but also non-SS crosslinking between gluten proteins. The decrease of free SH but also SS groups confirmed that the baking process led to non-SS crosslinks involving SH groups. Previous studies suggest the significance of lanthionine as non-SS crosslink of wheat gluten (Rombouts et al., 2016). Also, lower levels of total CSH and GSH induced by the baking process indicated the involvement of free SH compounds in this kind of non-SS crosslink formation. The losses of total CSH and GSH were dependent on the baking temperature. GSH loss occurred only in the crust samples, i.e. if samples were exposed to a very high temperature, CSH loss was highest in the crust samples. A decrease of total CSH and GSH after baking was described earlier by Reinbold (2011), who suggested free SH being hyperoxidized to their sulfinic and sulfonic acids. The levels of S-containing groups and compounds did not significantly differ between the baked samples. Thus, yet another type of non-SS crosslinks may be responsible for the lower gluten extractability of the pretzel crust samples. Previous studies have demonstrated the formation of lysinoalanine in pretzels (Sternberg et al., 1975; Raymond, 1980; Hasegawa et al., 1987; Rombouts et al., 2012). It is strongly favoured at higher pH conditions (Friedman, 1999). Obviously, also the presence of Maillard reaction derived crosslinks should be further elucidated in pretzel crust.

## 5 Conclusions

In conclusion, gluten protein extractability rather than the composition is crucial for gluten detection and quantitation by ELISA in baked goods. Here the focus needs to be on a complete extraction of both gliadins as well as glutenins. Because the formation of insoluble gluten polymers results not only from SS but also non-SS crosslinking during processing, none of the applied extraction solvents achieved a complete gluten extraction from the baked goods. The different baking conditions led to significantly reduced recovery rates (20 - 50%) of the gluten incurred products analysed by ELISA. Additional alkali treatment of the pretzel crust resulted in the lowest recoveries accompanied by a reduced glutenin extractability. Though in our study the conversion factor of gliadin to gluten for ELISA is sufficient to compensate the reduced extractabilities, the influence of processing of especially very low gluten foods should be considered in risk assessment and risk management decisions. Further studies are needed to gain more understanding of gluten crosslinking during processing to optimise gluten extraction methods and therefore accurate gluten detection and quantitation.

## Funding

We acknowledge support by the KIT-Publication Fund of the 10.13039/100009133Karlsruhe Institute of Technology.

## CRediT authorship contribution statement

**Tanja Miriam Schirmer:** Data curation, Formal analysis, Investigation, Methodology, Visualization, Writing – original draft. **Katharina Anne Scherf:** Conceptualization, Funding acquisition, Resources, Supervision, Writing – review & editing.

## Declaration of competing interest

The authors declare that they have no known competing financial interests or personal relationships that could have appeared to influence the work reported in this paper.

## Data Availability

Data will be made available on request.
